# The Association of the Triglyceride-to-HDL Cholesterol Ratio with Insulin Resistance in White European and South Asian Men and Women

**DOI:** 10.1371/journal.pone.0050931

**Published:** 2012-12-10

**Authors:** Samiul A. Mostafa, Melanie J. Davies, Danielle H. Morris, Tom Yates, Balasubramanian Thiagarajan Srinivasan, David Webb, Emer Brady, Kamlesh Khunti

**Affiliations:** 1 Department of Cardiovascular Sciences, University of Leicester, Leicester, United Kingdom; 2 Department of Health Sciences, University of Leicester, Leicester, United Kingdom; Mayo Clinic College of Medicine, United States of America

## Abstract

**Introduction:**

There is recent interest surrounding the use of the triglyceride-to-HDL cholesterol ratio as a surrogate marker of insulin resistance in clinical practice, as it may identify people at high risk of developing diabetes or its complications. However, it has been suggested using this lipid ratio may not be appropriate for measuring insulin resistance in African-Americans, particularly women. We investigated if this inconsistency extended to South Asian women in a UK multi-ethnic cohort of White Europeans and South Asians.

**Methods:**

Cross-sectional analysis was done of 729 participants from the ADDITION-Leicester study from 2005 to 2009. The association between tertiles of triglyceride-to-HDL cholesterol ratio to fasting insulin, homeostatic model of assessment for insulin resistance (HOMA1-IR), quantitative insulin sensitivity check index (QUICKI) and glucose: insulin ratio was examined with adjustment for confounding variables.

**Results:**

Incremental tertiles of the triglyceride-to-HDL cholesterol ratio demonstrated a significant positive association with levels of fasting insulin, HOMA1-IR, glucose: insulin ratio and a negative association with QUICKI in White European men (n = 255) and women (n = 250) and South Asian men (n = 124) (all p<0.05), but not South Asian women (n = 100). A significant interaction was demonstrated between sex and triglyceride-to-HDL cholesterol ratio tertiles in South Asians only (p<0.05). The area under the receiver operating characteristic curve for triglyceride-to-HDL cholesterol ratio to detect insulin resistance, defined as the cohort HOMA1-IR≥75^th^ percentile (3.08), was 0.74 (0.67 to 0.81), 0.72 (0.65 to 0.79), 0.75 (0.66 to 0.85) and 0.67 (0.56 to 0.78) in White European men and women, South Asian men and women respectively. The optimal cut-points for detecting insulin resistance were 0.9–1.7 in mmol/l (2.0–3.8 in mg/dl) for the triglyceride-to-HDL ratio.

**Conclusion:**

In South Asian women the triglyceride-to-HDL cholesterol ratio was not associated with insulin resistance; therefore there may be limitations in its use as a surrogate marker in this group.

## Introduction

The triglyceride-to-HDL cholesterol ratio has been investigated recently for various potential clinical uses in adult and paediatric populations [1]–[12]. Previous research has demonstrated its positive associations with adverse cardio-metabolic risk factor profiles, metabolic syndrome and prediction of incident diabetes or its complications [1]–[12]. This may occur as the triglyceride-to-HDL cholesterol ratio demonstrates an association with insulin resistance [7], [9]–[12]. Therefore, it may form a convenient method of estimating levels of insulin resistance in comparison to time consuming glucose clamp techniques. However the association between the lipid ratio and insulin resistance is not consistent in African-Americans, particularly women [7], [13]–[16]. A similar non-significant association has been reported in a small study of South Asians (people of Indian, Pakistani and Bangladeshi origin) but that study did not assess findings by sex [17]. We wished to assess if the potential problem in African-American women extended to South Asian women or if the triglyceride-to-HDL cholesterol ratio was consistent in both sexes.

South Asians form over one-fifth of the world's population, including over 4 million migrants living in both the US and UK [18]–[19]. There are consistent reports of high levels of diabetes, prediabetes and coronary heart disease in South Asians, which is attributed predominantly to increased levels of insulin resistance [20]–[23]. Therefore the triglyceride-to-HDL cholesterol ratio could form a valuable clinical tool in this ethnic group. The aim of this study was to investigate the association between triglyceride-to-HDL cholesterol ratio and markers of insulin resistance in a western multi-ethnic cohort of White European and South Asian men and women. Secondly, we wished to compare performance of the triglyceride-to-HDL ratio for detecting insulin resistance to triglyceride alone.

## Methods

### Design overview, setting and participants

The analysis was performed using data from the ADDITION-Leicester study (Anglo-Danish-Dutch Study of Intensive Treatment in People with Screen Detected Diabetes in Primary Care, registration trial number NCT00318032). ADDITION-Leicester consisted of a population based screening program to identify individuals with diabetes who then underwent a five year cluster randomised controlled trial. This study contributed to the multi-centre ADDITION-Europe, but was also a stand-alone study designed to investigate differences between westernised South Asians and White Europeans [24]. Here we focus on cross-sectional screening data from participants in the UK based Leicester arm only. A description of study design and methods has been reported elsewhere in detail [24]. Briefly, a random sample of individuals aged 40 to 75 years were invited via Leicestershire community practices to attend a screening test between 2005 and 2009. Following an overnight fast for at least eight hours, participants underwent a 75 g oral glucose tolerance test, diagnosed according to World Health Organisation diagnostic criteria 1999, and had selected cardiovascular risk factors measured [25]. Participants were asked to fill out questionnaires including demographic information on age, sex and ethnic group. White European and South Asian ethnic groups were defined according to UK national census categories [26]. South Asians were a mixture of first and second generation immigrants. Socio-economic deprivation was calculated using the Index of Multiple Deprivation which assesses for income, employment, education and living environment [27]. The specific sub-cohort studied in this analysis came from a proportion of stored blood samples taken during baseline screening, which were measured for insulin levels (n = 892). We specifically over-selected insulin samples from glucose intolerant participants [diabetes, impaired fasting glucose and/or impaired glucose tolerance using WHO 1999 criteria, 25] as these were of interest and achieved better numerical parity with samples from normal glucose regulation participants. Beyond this stratification, all insulin samples were randomly selected and maintained an equivalent proportion of White Europeans and South Asians to the total cohort. There were no participants with a serum creatinine value greater than 140 µmol/l, taking thyroid medications or with a self-reported history of liver disease. Furthermore, participants on lipid lowering therapy (n = 157) or oral corticosteroids (n = 6) were excluded. From the remaining 729 individuals, 40 (15.6%) and 29 (11.6%) white European men and women respectively were detected as having diabetes, while these values were 19 (15.3%) and 13 (13%) in South Asians men and women. The corresponding values for glucose intolerance were 129 (50.6%) and 122 (48.8%) in White European men and women, with 54 (43.5%) and 50 (50%) in South Asian men and women. There were no significant differences in people with diabetes or glucose intolerance between these groups. Anthropometric measurements were taken in accordance with standardised operating procedures by trained staff members. Height was measured to the nearest 0.1 cm with a rigid stadiometer. Body weight was measured in light indoor clothing to the nearest 0.1 kg using a Tanita scale (Tanita, Europe). Brachial blood pressure was measured three times using standardised Omron M7 digital sphygmomanometers (Omron Healthcare, Milton Keynes, UK). The average of the second and third blood pressure readings was recorded. All participants provided written informed consent. Ethical approval was obtained from the Leicestershire, Northampton and Rutland Research Ethics Committee.

### Laboratory assays

Fasting lipid levels were collected in lithium heparin bottles and measured on a Dade Behring Dimension analyser, Newark, USA. HDL cholesterol was measured after isolation of other non HDL apolipoproteins; triglycerides were measured following enzymatic hydrolysis to glycerol. Glucose samples were collected in fluoride oxalate tubes and measured on an Abbott Aeroset clinical chemistry (Abbott laboratories, Maidenhead, UK) analyser. Glucose and lipid profiles were measured on the same day in the same laboratory with quality control assessments conducted daily. Fasting insulin levels were collected in lithium heparin tubes and centrifuged immediately, before stored in a −80 degrees Celsius freezer. Insulin samples were analysed on a Perkin Elmer time-resolved fluoro-immuno assay on an Auto DELFIA, which has less than 1% cross-reactivity with C-peptide and proinsulin. The inter- and intra-coefficient of variations were less than 2.5% for insulin, HDL, triglycerides and glucose.

### Statistical analysis

SPSS version 18.0 (SPSS Inc., Chicago, IL, USA) was used to perform statistical analysis, except that XLSTAT version 2011.5 (Addinsoft, Paris, France) was used for receiver operating characteristic (ROC) curve analysis. Baseline demographics of the analysed population were reported stratified by ethnic group and sex. The distribution of continuous variables was inspected for any outlying values, skewness and kurtosis. Means and standard deviations were reported for normally distributed variables. Non-normally distributed parameters were logarithmically transformed and results expressed as geometric mean with 95% confidence intervals. Discrete variables were analysed using chi-squared tests. A high triglyceride level was defined as ≥1.7 mmol/l and low HDL level as <1.0 mmol/l and <1.3 mmol/l in men and women respectively [28].

Within each sex and ethnic group, the triglyceride-to-HDL cholesterol ratio was divided into thirds (tertiles) and tested for association with markers of insulin resistance using analysis of covariance modelling with adjustment for confounding variables. Insulin resistance was assessed using the following four markers. Homeostasis model assessment of insulin resistance (HOMA1-IR) was calculated as fasting glucose (mmol/l)×fasting insulin (µIU/ml)/22.5 which correlates well with insulin resistance [29]. A HOMA1-IR value above 75^th^ percentile was defined as insulin resistance, following similar use in other studies [16]. A second definition of insulin resistance consisted of a fasting insulin level above 75^th^ percentile (hyperinsulinaemia) in people without diabetes [9], [30]. Thirdly, quantitative insulin sensitivity check index (QUICKI) represents a strong measure of insulin sensitivity and was calculated using the formula: 1/(log insulin, µIU/ml, +log glucose, mmol/l) [31]. Finally, the fasting glucose: fasting insulin ratio, a good marker of insulin sensitivity, was calculated [32]. The covariates included in the model were selected from potential confounders using a backward elimination process with variables removed if they were not significant at the 5% level. Using this method, age, systolic blood pressure, body mass index and LDL-cholesterol were included in the model, while deprivation level, diastolic blood pressure, waist circumference and creatinine were excluded. The analysis was repeated with an interaction between sex and triglyceride-to-HDL cholesterol ratio tertiles after pooling male and female datasets in each ethnic group. Models were checked for absence of collinearity of variables using variance inflation factor and normality of residuals was tested with Shapiro-Wilk tests.

Finally, we analysed performance of the triglyceride-to-HDL cholesterol ratio to detect insulin resistance, as measured by the cohort HOMA-IR≥75^th^ percentile (3.08), by calculating the area under the receiver operating characteristic (AUROC) curve and its 95% confidence intervals [33]. An AUROC>0.7 is generally considered an acceptable test performance whereas <0.7 is an indication of weaker and unacceptable performance [34]. The optimal cut-point was calculated as the best balance in a trade-off between sensitivity and specificity, using maximal values derived from the Youden Index (sensitivity+specificity−1) [35]. A two-sided p-value of <0.05 was considered statistically significant.

## Results

Baseline demographics of the cohort investigated are shown in Table 1. In both ethnic groups, women presented with a lower mean height, weight, waist circumference, diastolic blood pressure, creatinine, triglyceride-to-HDL cholesterol ratio and higher mean HDL level than men. Additionally in the White European group, women (n = 250) demonstrated a lower mean fasting plasma glucose, HOMA1-IR and higher mean age and QUICKI level compared to men (n = 255). Whereas in the South Asian group, women (n = 100) presented with a lower mean systolic, total and LDL-cholesterol, triglyceride, non-HDL cholesterol and higher body mass index compared to men (n = 124).

**Table 1:** Selected baseline demographics of ADDITION-Leicester cohort investigated, separated by ethnicity and sex.

	White Europeans (n = 505)		South Asian (n = 224)	
	Men (n = 255)	Women (n = 250)	Men (n = 124)	Women (n = 100)
Age (years)	60.4 (9.0)	62.1 (8.9)†	52.2 (10.4)	52.1 (9.9)
Deprivation level score	14.7 (13.5–16.0)	15.1 (13.8–16.5)	19.6 (16.9–25.3)	21.0 (14.1–26.7)
Height (m)	1.75 (0.1)	1.60 (0.1)*	1.69 (0.1)	1.54 (0.1)*
Weight (kg)	90.9 (15.2)	77.6 (15.0)*	77.0 (12.2)	72.1 (14.3)‡
Body Mass Index (kg/m^2^)	29.5 (4.2)	30.1 (5.5)	27.0 (3.6)	30.2 (5.4)*
Waist Circumference (cm)	104.0 (11.2)	94.3 (13.6)*	96.4 (9.3)	92.7 (12.3)†
Systolic blood pressure (mmHg)	143.9 (18.1)	141.9 (20.5)	140.6 (18.0)	133.5 (21.9)†
Diastolic blood pressure (mmHg)	88.7 (9.5)	85.8 (10.9)‡	88.7 (11.3)	85.2 (10.8)†
Creatinine (µmol/l)	94.0 (11.7)	79.1 (11.2)*	94.7 (13.8)	74.1 (8.8)*
Triglycerides (mmol/l)	1.4 (1.3–1.5)	1.3 (1.2–1.4)	1.5 (1.3–1.6)	1.3 (1.1–1.4)†
HDL cholesterol (mmol/l)	1.2 (1.2–1.3)	1.5 (1.4–1.5)*	1.2 (1.1–1.2)	1.3 (1.2–1.3)†
Triglyceride-to-HDL-Cholesterol ratio	1.2 (1.1–1.3)	0.9 (0.9–1.0)*	1.3 (1.1–1.4)	1.0 (0.9–1.1)†
LDL cholesterol (mmol/l)	3.8 (0.9)	3.9 (1.0)	3.6 (0.9)	3.2 (0.7)*
Fasting plasma glucose (mmol/l)	5.9 (1.8)	5.5 (1.1)‡	5.8 (1.5)	5.5 (1.4)
2 hour plasma glucose (mmol/l)	7.6 (4.0)	7.6 (3.4)	8.0 (4.5)	7.8 (3.3)
Fasting Insulin (µIU/ml)	7.6 (7.0–8.2)	6.9 (6.4–7.5)	8.7 (7.8–9.8)	8.7 (7.7–9.7)
HOMA1-IR	1.9 (1.7–2.1)	1.7 (1.5–1.8)‡	2.2 (1.9–2.5)	2.1 (1.8–2.4)
QUICKI	0.62 (0.61–0.64)	0.65 (0.63–0.67)‡	0.60 (0.58–0.63)	0.61 (0.59–0.63)
Glucose: Insulin ratio				
% High triglyceride ≥1.7 mmol/l	33.3	30.0	40.3	22.4‡
% Low HDL	11.4	24.0*	16.9	48.0*

Continuous variables presented as mean (standard deviation) or geometric mean (95% confidence intervals) after initial log transformation of non-normally distributed variables. Low HDL defined <1.0 and 1.3 mmol/l in males and females respectively.

* p<0.001,

‡ p<0.01,

† p<0.05.

Incremental tertiles of the triglyceride-to-HDL cholesterol ratio in White European women and men and South Asian men demonstrated a significant positive association with insulin level, hyperinsulinaemia, HOMA1-IR level and HOMA1-IR≥75^th^ percentile and a negative association with QUICKI (p-values in Table 2). However, based on smaller numbers, in South Asian women there were no significant associations. When men and women in each ethnic group were pooled, the analyses indicated a significant interaction between triglyceride-to-HDL cholesterol ratio and sex in South Asians on insulin (p = 0.02), HOMA-IR (p = 0.04) and QUICKI (p = 0.03). The same analyses in White Europeans revealed no significant interactions (all p>0.2).

**Table 2:** The Association of triglycerides-to-HDL ratio (THR) tertiles with markers of insulin resistance (IR).

		White European		South Asian	
Insulin marker	Tertile of THR	Men	Women	Men	Women
Insulin, µIU/ml	Lowest	6.8 (6.1–7.7)	5.7 (5.1–6.4)	6.7 (5.6–8.0)	8.7 (6.9–10.9)
	Middle	7.2 (6.5–8.0)	6.7 (5.9–7.5)	9.3 (7.9–11.1)	8.0 (6.5–9.8)
	Highest	9.1 (8.1–10.3)‡	8.1 (7.1–9.1)	10.6 (8.9–12.6)‡	10.0 (7.8–12.7)
Hyperinsulinaemia, %	Lowest	14.3	6.3	5.1	6.7
	Middle	17.3	13.7	20.0	16.1
	Highest	36.1‡	30.9	35.5‡	25.0
HOMA1-IR	Lowest	1.7 (1.5–1.9)	1.4 (1.2–1.5)	1.7 (1.4–2.0)	2.0 (1.6–2.6)
	Middle	1.8 (1.6–2.0)	1.6 (1.4–1.8)	2.3 (1.9–2.8)	1.8 (1.5–2.4)
	Highest	2.5 (2.2–2.8)*	1.9 (1.7–2.2)‡	2.7 (2.2–3.3)‡	2.4 (1.9–3.2)
HOMA1-IR≥75^th^ percentile, %	Lowest	14.6	9.6	12.2	15.2
	Middle	13.8	24.1	19.5	21.9
	Highest	46.5†	41.7*	42.9‡	39.4
QUICKI	Lowest	0.65 (0.62–0.67)	0.69 (0.66–0.72)	0.65 (0.62–0.69)	0.62 (0.58–0.66)
	Middle	0.64 (0.62–0.66)	0.65 (0.62–0.68)	0.59 (0.56–0.62)	0.63 (0.59–0.67)
	Highest	0.58 (0.56–0.6)*	0.63 (0.6–0.65)†	0.57 (0.54–0.60)‡	0.56 (0.52–0.61)
Glucose: Insulin ratio	Lowest	0.83 (0.74–0.94)	0.92 (0.82–1.03)	0.84 (0.71–1.00)	0.66 (0.55–0.79)
	Middle	0.77 (0.69–0.85)	0.82 (0.74–0.92)	0.61 (0.52–0.72)	0.65 (0.55–0.78)
	Highest	0.67 (0.75–0.59)†	0.62 (0.56–0.70)*	0.53 (0.45–0.63)‡	0.53 (0.44–0.63)

The cut-points for tertiles of THR were as follows. White European men: <0.83, 0.83 to 1.50, ≥1.51; White European women: <0.67, ≥0.67 to <1.14, ≥1.14; South Asian men: <0.91, ≥0.91 to <1.70, ≥1.70; South Asian women: <0.73, ≥0.73 to <1.18, ≥1.18. Continuous variables presented as geometric mean (95% confidence intervals) including adjustments for age, BMI, LDL-cholesterol and SBP. P-values for trend across the tertiles:

* p<0.001,

‡ p<0.01,

† p<0.05.

Key: HOMA1-IR = Homeostasis model assessment of insulin resistance, IR = Insulin resistance, QUICKI = quantitative insulin sensitivity check index THR = triglyceride-to-HDL ratio.

**Figure 1:**
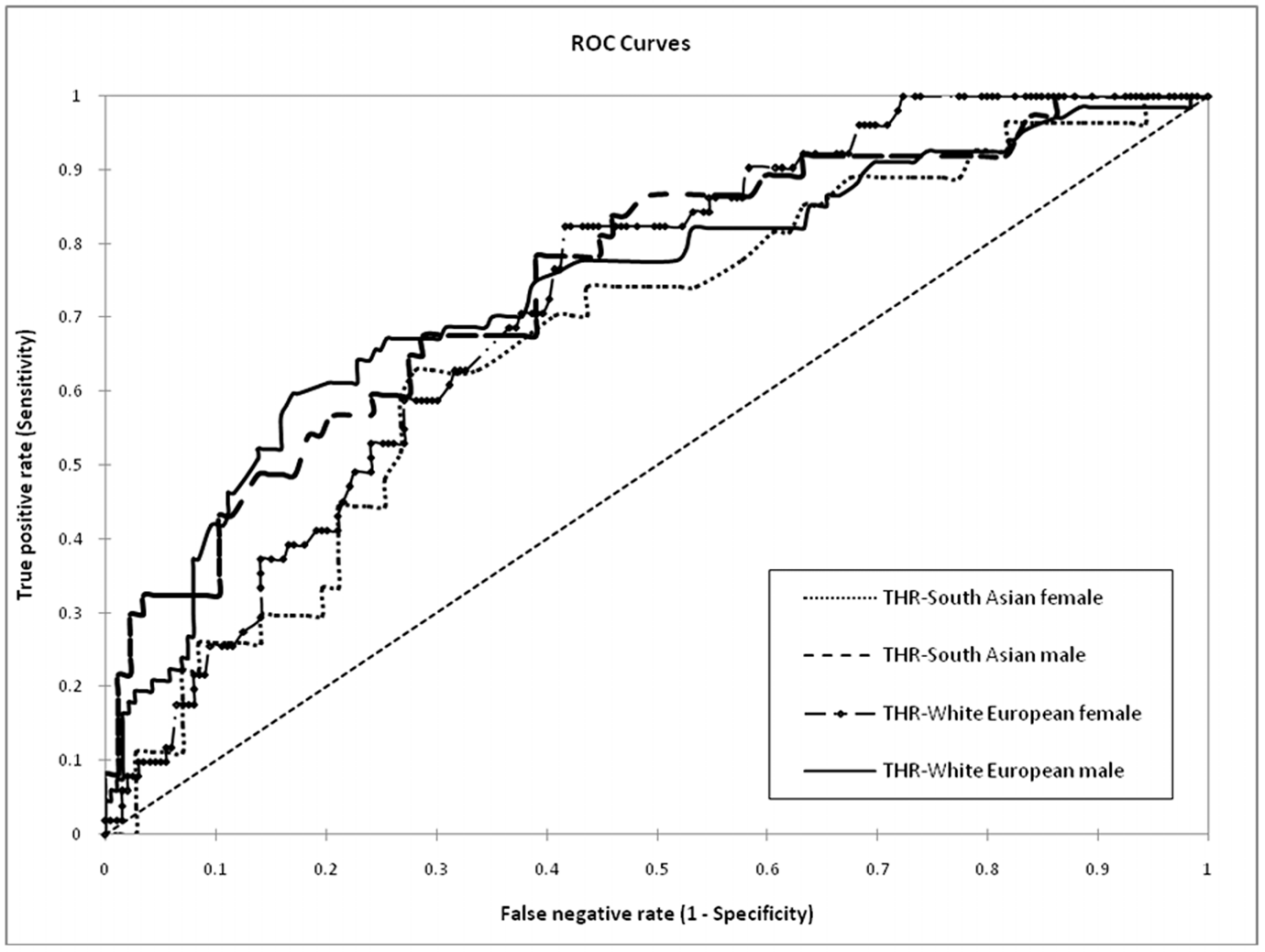
Receiver operating characteristic curves of the triglyceride-to-HDL cholesterol ratio (THR) to detect insulin resistance, defined as cohort HOMA-IR≥75th percentile, in White European and South Asian men and women.

The AUROC curve for triglyceride-to-HDL cholesterol ratio to detect insulin resistance was 0.73 (95% confidence intervals 0.65 to 0.80), 0.71 (0.64 to 0.78), 0.74 (0.62 to 0.83) and 0.68 (0.56 to 0.80) in White European men and women, South Asian men and women respectively (figure 1). For triglyceride alone, the corresponding values were 0.71 (0.64 to 0.78), 0.68 (0.630 to 0.74), 0.73 (0.64 to 0.82) and 0.62 (0.51 to 0.74) respectively. The optimal triglyceride-to-HDL cholesterol ratio cut-points for detecting insulin resistance were 1.7 and 0.9 and in mmol/l SI unit (equivalent of 3.8 and 2.0in mg/dl conventional unit) in White European men and women respectively; whilst in South Asian men and women these were 1.2 and 1.1 in mmol/l (2.8 and 2.5 in mg/dl) respectively. When the same analysis was performed for triglyceride the optimal cut-points were 1.8 and 1.4 mmol/l (159 and 124 mg/dl) in White European men and women; while in South Asian men and women these were both 1.6 mmol/l (142 mg/dl) respectively.

## Discussion

The main findings of this study demonstrate that the triglyceride-to-HDL cholesterol ratio is associated with insulin resistance measures in White Europeans and South Asian men; this relationship appeared to be less established in South Asian women. However the test performance of triglyceride-to-HDL cholesterol ratio to detect insulin resistance was variable, with the 95% confidence intervals of the area under the ROC curve falling below the acceptable range in all four groups tested. Finally the optimal cut-points for triglyceride-to-HDL cholesterol ratio and triglyceride to detect insulin resistance were 0.9 to 1.7 and 1.4 to 1.8 mmol/l respectively.

Previous studies have reported the triglyceride-to-HDL cholesterol ratio is a valid marker of insulin resistance, particularly in White populations [7], [9], [11]–[12]. However in African-Americans this lipid ratio can demonstrate a weak or non-significant association with insulin resistance, particularly in women [7], [13]–[16]. The AUROC to detect the upper quartile of HOMA-IR in African-American women was 0.66 compared to 0.77 in African-American men [16]. African-Americans produce high levels of the enzyme lipoprotein lipase clearing triglyceride-rich lipoproteins from blood circulation, even in insulin resistant states [36]. Therefore a relatively lower triglyceride level is maintained. In Hispanic populations, the association between the lipid ratio and insulin resistance needs further clarification as studies generally not stratified by sex show opposite findings [7], [9]–[10]. Only one previous study in South Asians has analysed the relationship between the triglyceride-to-HDL cholesterol ratio with a single insulin resistance marker and failed to show an association [17]. However the results were not stratified by sex. Our study demonstrated a significant association in South Asian men but not women, which was also suggested by a significant sex interaction. Therefore, this appears to demonstrate a similar result to African-Americans. Why this occurs is not fully understood. It may involve the relatively high proportion of South Asian women (48%) classified as having a low HDL cholesterol level compared to White European women (24%, Table 1). Also the relative proportion of HDL subtypes is known to vary between these ethnic groups [37].

Despite our novel finding, we acknowledge the number of South Asian women in the present study was limited to 100 and therefore results may be somewhat prone to power issues. The percentage of South Asian women with insulin resistance defined by fasting insulin and HOMA-IR values in the upper quartile appeared to show increasing trend across incremental triglyceride-to-HDL cholesterol ratio tertiles but neither result achieved statistical significance. Therefore our results should be considered as hypothesis generating and investigated further in well-designed prospective studies. Furthermore, other potential confounding factors cannot be accounted for which may have influenced our results. These include physical activity levels and dietary intake, including alcohol consumption. Also, our South Asian population was a mix of first and second generation immigrants. Finally, medications which affect insulin or HDL-cholesterol levels, such as hormone replacement therapy/post-menopausal oestrogens, were not known as only cardiovascular medications were electronically recorded in ADDITION-Leicester databases. However South Asian women were not over-represented in exclusions made due to lipid lowering therapies; also there were no exclusions made due to out-lying insulin values in South Asian women.

We acknowledge the HOMA-IR values in the present study may appear to be lower than in some, but not all cohorts investigating the same topic [1], [10], [16]–[17]. One explanation may be the lack of an international standardisation of insulin assays, with the possibility of large inter-assay variation [38]–[39]. Also most epidemiology studies use single insulin samples to calculate HOMA-IR values, possibly introducing further variation [39].

There are a number of strengths of this study. We investigated a multi-ethnic cohort of White Europeans and South Asians, where all participants underwent phenotyping as well insulin measurements. Secondly, we reduced the large intra-individual variation of triglyceride by using fasting samples. Thirdly, the results were stratified by sex and tested for an interaction effect with triglyceride-to-HDL cholesterol ratio. Regarding potential limitations, we were unable to measure insulin resistance directly in this cohort of 729 individuals, a recognised problem in similar studies [9]–[10], [12], [14]–[15], [17]. However we assessed four different validated or recommended markers of insulin resistance/sensitivity to reduce bias and inaccuracies from any one single marker. Secondly, lipid profiles and insulin levels were measured using single venous samples.

Our study demonstrated the optimal cut-points for the triglyceride-to-HDL cholesterol ratio to detect insulin resistance were 1.7 and 0.9 in mmol/l international (SI) unit (or 3.8 and 2.0 in mg/dl conventional unit) for White men and women, and 1.2 and 1.1 mmol/l (2.8 and 2.5 mg/dl) for South Asian men and women. Previous studies have demonstrated the same optimal cut-points to detect insulin resistance are between 2.0 to 2.5 mg/dl in African-Americans and 3.0 mg/dl in both non-Hispanic Whites and Mexican-Americans without diabetes. Using international units, a separate study has reported optimal cut-points for detecting insulin resistance of 0.9, 1.1, 1.1 and 1.8 mmol/l for Aboriginal, Chinese, European and South Asian populations. The reported differences between optimal cut-points in various studies may represent a mix of ethnic differences and/or different methods employed, such as measuring insulin resistance directly or using HOMA-IR models.

### Potential implications for clinical practice

The potential translational value of the triglyceride-to-HDL cholesterol ratio into clinical practice has yet to be determined. Triglycerides and HDL-cholesterol are included within diagnostic criteria of the metabolic syndrome separately. A combined lipid ratio may better reflect the overall interaction between lipid/lipoprotein fractions, and therefore associations with insulin resistance [40]. Our study showed performance of triglyceride-to-HDL ratio to detect insulin resistance was stronger than either triglyceride alone. However the 95% confidence intervals of AUROCs suggested a sub-optimal performance in all sub-groups investigated. Furthermore, our study and others have demonstrated the triglyceride-to-HDL cholesterol ratio may not associate with insulin resistance in women from two ethnic minority groups [7], [16].

To our knowledge this is the first study to demonstrate the optimal cut-points for triglyceride level detecting insulin resistance in South Asians are lower than those used in the metabolic syndrome, also termed the insulin resistance syndrome (28). International organisations have recommended using lower ethnic specific cut-points for waist circumference and body mass index in the metabolic syndrome for South Asians in response to high levels of diabetes [41]–[42]. Future studies should explore if recommendations on lower ethnic specific triglyceride cut-points are required in South Asians. In summary, further evidence of triglyceride-to-HDL cholesterol ratio is required to determine its utility as a surrogate marker of insulin resistance in well-designed prospective studies, which may require stratification of results by sex when ethnic minority groups are investigated.
